# Divergent TLR2 and TLR4 Activation by Fungal Spores and Species Diversity in Dust from Waste Sorting Plants

**DOI:** 10.1128/aem.01734-22

**Published:** 2023-03-01

**Authors:** Anani K. Afanou, Sunil Mundra, Eva Lena Fjeld Estensmo, Ine Pedersen, Jens Rasmus Liland, Elke Eriksen, Pål Graff, Tonje Trulssen Hildre, Karl-Christian Nordby, Anne Straumfors

**Affiliations:** a National Institute of Occupational Health (STAMI), Oslo, Norway; b Department of Biology, College of Science, United Arab Emirates University, Al-Ain, Abu-Dhabi, United Arab Emirates; c Khalifa Center for Genetic Engineering and Biotechnology, United Arab Emirates University, Al Ain, United Arab Emirates; d Univeristy of Oslo, Department of Bioscience, Oslo, Norway; e Oslo University Hospital, Department of Lung Medicine, Oslo, Norway; Chalmers University of Technology

**Keywords:** waste workers, bioaerosols, mycobiome, TLR2 and TLR4

## Abstract

This manuscript presents the results of an exploratory study on the relationships between NF-κB response through Toll-like receptor (TLR) activation by dust characterized by fungal spore concentrations and species diversity. Personal total dust samples were collected from Norwegian waste sorting plants and then characterized for fungal spores and fungal species diversity, as well as for other bioaerosol components, including endotoxins and actinobacteria. The ability of the dust to induce an NF-κB response by activating TLR2 and TLR4 *in vitro* was evaluated, as well as the relationship between such responses and quantifiable bioaerosol components. The average concentrations of bioaerosols were 7.23 mg total dust m^−3^, 4.49 × 10^5^ fungal spores m^−3^, 814 endotoxin units m^−3^, and 0.6 × 10^5^ actinobacteria m^−3^. The mean diversity measurements were 326, 0.59, and 3.39 for fungal richness, evenness, and Shannon index, respectively. Overall, fungal operational taxonomic units (OTUs) belonging to the Ascomycota phylum were most abundant (55%), followed by Basidiomycota (33%) and Mucoromycota (3%). All samples induced significant NF-κB responses through TLR2 and TLR4 activation. While fungal spore levels were positively associated with TLR2 and TLR4 activation, there was a trend that fungal species richness was negatively associated with the activation of these receptors. This observation supports the existence of divergent immunological response relationships between TLR activation and fungal spore levels on one hand and between TLR activation and fungal species diversity on the other. Such relationships seem to be described for the first time for dust from waste facilities.

**IMPORTANCE** This manuscript presents results on multifactorial characterization of bioaerosol exposure in Norwegian waste sorting plants and the potential of such airborne dust to induce NF-κB reactions through TLR2 and TLR4 activations in an *in vitro* reporter cell model system. Our data revealed that increasing fungal spore levels in the dust is associated with increased activation of TLR2 and TLR4, whereas increasing fungal OTU richness is associated with decreasing activation of these receptors. The NF-κB-induced responses by the collected dust represent, therefore, effective measures of potential key immunological effects induced by a complex mixture of hazardous components, including characterized factors such as endotoxins, fungal spores, bacteria, and many other uncharacterized components. The key immunological events reported here are suggested as holistic alternatives to today’s bioaerosol exposure characterization approaches for epidemiological studies in the future.

## INTRODUCTION

A variety of operations in waste processing can generate significant levels of complex microbial-rich dust with relatively high fractions of fungal particles. Although fungal exposure has been described for such occupational settings as a potent inflammatory stimulus, the relationships with inflammatory markers in workers are often inconsistent (1–5) except for specific cases with Aspergillus spp. (6).

The airborne mycobiome from waste processing plants encompasses wide taxonomic diversity (7–13), as well as structural and metabolic components that can interact synergistically or antagonistically to elicit immune responses, as reviewed for farming settings by May et al. (14). The use of adequate *in vivo* or *in vitro* models is paramount for identifying hazards and predicting the risks associated with such complex exposure (15).

Many *in vitro* cell model studies focusing on proinflammatory responses and cytotoxicity have provided valuable insight into our understanding of biologically relevant responses initiated by dust from various exposure settings (6, 16–27). However, the underlying pathways and mechanisms of these responses remain unexplored. In fact, many triggering and underlying mechanisms begin with the activation of Toll-like receptors (TLRs), which are well-described pattern recognition receptors (PRRs) of the innate immune system (28). These receptors play a key role in the recognition of microbe-associated molecular patterns (MAMPs) and triggering the internalization and the destruction of microbial particles by immune cells. Various fungal cell wall components such as mannoproteins, beta-glucans, membrane proteins, and chitin can induce inflammatory responses in the respiratory tract. While such responses are critical for training the immune system against MAMPs, chronic exposure with repetitive cycles of oxidation and inflammation in the airways is associated with chronic airway disease and a decline in lung function over time (26, 29).

Relatively high exposure levels of fungi have been measured in waste management by the use of personal (24) and stationary sampling methods (5, 25, 30–32). Exposure hazard characterization focusing on how fungal spores and species diversity, together with other common bioaerosol components, activate TLR2 and TLR4, known as innate immune system sentinel receptors (33), may provide new insights into important biological effects associated with worker exposure during waste treatment processes. In the present study, we sought to explore how fungal species diversity or fungal spore levels in dust collected during recycling processes of cardboards, plastics, fillers, gypsum boards, old windows, wooden furniture, and garden organic waste materials influence the activation of these receptors. We aimed, therefore, to (i) quantify the personal exposure to fungi, including fungal spores and species diversity, as well as endotoxins and actinobacteria; (ii) characterize the induction of the NF-κB response *in vitro* through TLR2 and TLR4 activation; and (iii) explore the relationship between exposure parameters, fungal species diversity, and the cell-based immunological effects.

## RESULTS

### Exposure levels of dust, fungal spores, actinobacteria, and endotoxins.

**FIG 1:**
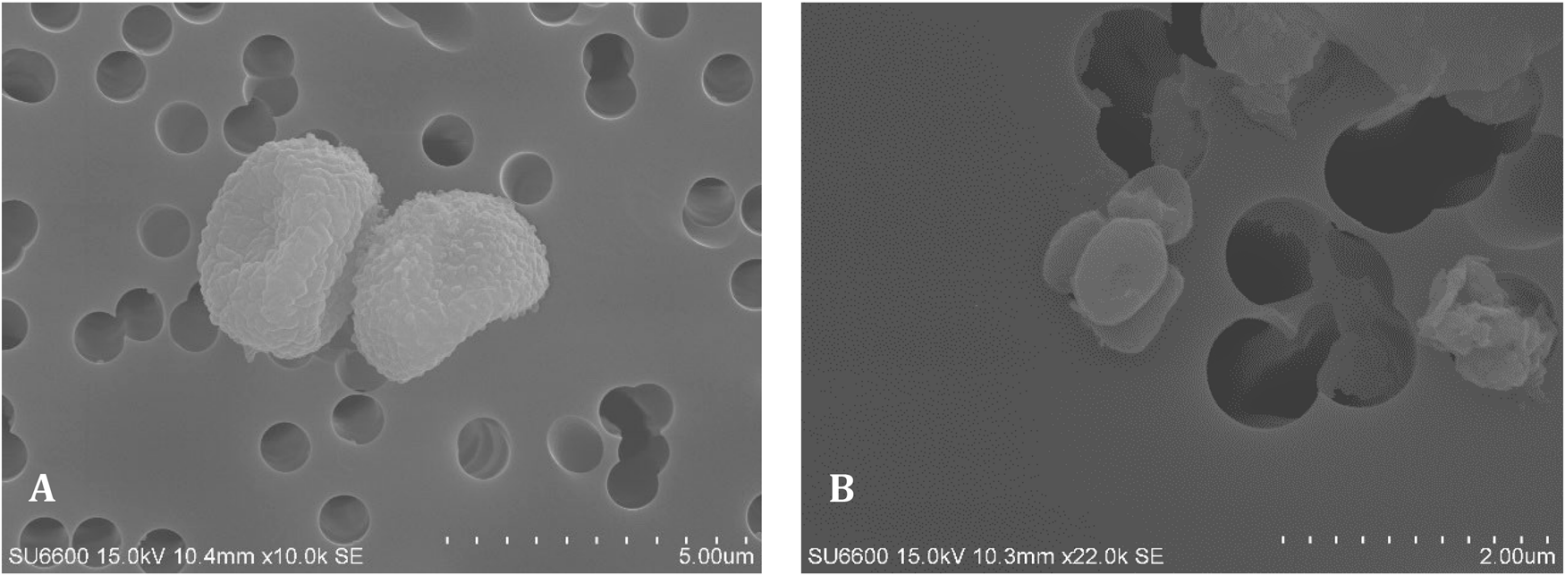
Micrographs of spores from fungi (A) and actinobacteria (B).

Workers were exposed to dust concentrations ranging from 0.31 mg m^−3^ to 50.22 mg m^−3^ with a geometric mean (GM) and geometric standard deviation (GSD; in parentheses) of 1.8 (4.6) mg m^−3^. The arithmetic mean (AM) of dust exposure levels varied significantly between companies (*P* = 0.009), with the highest value measured in company 1 (AM [median], 13.35 [1.55] mg m^−3^) compared to company 4, with the lowest concentrations (0.40 [0.36] mg m^−3^) (Table 1). With the exception of 3 workers in company 1, who were exposed to 11.04, 48.55, and 50.22 mg m^−3^, respectively, the overall dust exposure values were below the Norwegian occupational exposure limit (OEL) of 5 mg m^−3^. The total dust contained relatively high levels of fungal spores (Fig. 1A) ranging from below the detection limit to 15.3 × 10^5^ spores m^−3^. Spore concentrations varied significantly between companies (*P* = 0.009) with the highest measured exposure levels at company 1 (AM [median], 7.76 (7.45) ×10^5^ spores m^−3^) compared to company 3 (0.4 [0.04] × 10^5^ spores m^−3^) (Table 1). Airborne actinobacteria were also detected but only in company 1 (Fig. 1B; Table 1) in 4 samples. Relatively high levels of endotoxins were detected in all samples, with only 3 samples having values below the recommended health-related OEL of 90 endotoxin units (EU) m^−3^ (34). Four workers were exposed to more than 1,000 EU m^−3^, while the exposure levels for the remaining workers varied between 90 and 1,000 EU m^−3^. The highest values were found in company 1 (AM [median], 1,466 [344] EU m^−3^) versus company 2, with the lowest levels (123 [126] EU m^−3^), but no statistically significant differences could be found between the companies (Table 1).

**TABLE 1:** Descriptive statistics of data overall and stratified by company^*e*^

Bioaerosol component	No.	AM	SD	Median	GM	GSD	Min	Max
Dust (mg m^−3^)								
Overall	18	7.23	15.54	1.44	1.79	4.56	0.31	50.22
Company 1	9	13.35	20.69	1.55^*a*^	3.98	5.18	0.8	50.22
Company 2	3	2.22	0.83	1.8	2.12	1.42	1.68	3.17
Company 3	3	0.73	0.52	0.54	0.62	2.01	0.34	1.33
Company 4	3	0.4	0.12	0.36^*a*^	0.39	1.34	0.31	0.54
Fungal spores (no. m^−3^)								
Overall	18	4.9 × 10^5^	4.6 × 10^5^	3 × 10^5^	1.95 × 10^5^	7.14	<LOD	15.3 × 10^5^
Company 1	9	7.76 × 10^5^	4.52 × 10^5^	7.45 × 10^5^^*b*^	6.28 × 10^5^	2.15	1.56 × 10^5^	15.3 × 10^5^
Company 2	3	3.19 × 10^5^	3.43 × 10^5^	2.67 × 10^5^	0.89 × 10^5^	15.7	<LOD	6.85 × 10^5^
Company 3	3	0.42 × 10^5^	0.67 × 10^5^	0.039 × 10^5^^*b*^	0.12 × 10^5^	7.25	<LOD	1.2 × 10^5^
Company 4	3	2.24 × 10^5^	1.09 × 10^5^	2.34 × 10^5^	2.04 × 10^5^	1.75	1.1 × 10^5^	3.28 × 10^5^
Actinobacteria (no. m^−3^)								
Overall	18	0.6 × 10^5^	1.4 × 10^5^	0.04 × 10^5^	0.091 × 10^5^	5.43	<LOD	5.6 × 10^5^
Company 1	9	1.08 × 10^5^	1.8 × 10^5^	0.053 × 10^5^	0.21 × 10^5^	8.31	<LOD	5.56 × 10^5^
Company 2	3	0.041 × 10^5^	0.0037 × 10^5^	0.039 × 10^5^	0.041 × 10^5^	1.09	<LOD	0.045 × 10^5^
Company 3	3	0.039 × 10^5^	0.01 × 10^5^	0.39 × 10^5^	0.39 × 10^5^	1.03	<LOD	0.4 × 10^5^
Company 4	3	0.37 × 10^5^	0.025 × 10^5^	0.37 × 10^5^	0.37 × 10^5^	1.07	<LOD	0.4 × 10^5^
Endotoxins (EU m^−3^)								
Overall	18	814	1,441	231	193	8.7	<LOD	5,321
Company 1	9	1,466	1,855	344	283	20.7	0.35	5,321
Company 2	3	123	22	126	121	1.2	99	143
Company 3	3	180	131	141	150	2.11	73	326
Company 4	3	183	198	77	124	2.84	60	411
Fungal OTU richness								
Overall	18	326	124	282	306	1.4	152	574
Company 1	9	276^*c*^	41	262	273	1.2	224	340
Company 2	3	303	107	266	291	1.4	219	424
Company 3	3	270	122	263	251	1.62	152	396
Company 4	3	557^*c*^	23	565	556	1.04	531	574
Evenness index								
Overall	18	0.59	0.12	0.60	0.57	1.31	0.23	0.76
Company 1	9	0.56	0.06	0.56	3.11	1.12	0.42	0.64
Company 2	3	0.61	0.04	0.60	3.47	1.14	0.58	0.66
Company 3	3	0.51	0.25	0.62	2.54	2.01	0.23	0.70
Company 4	3	0.72	0.03	0.71	4.55	1.04	0.70	0.76
Shannon diversity index								
Overall	18	3.39	0.83	3.36	3.261	1.367	1.14	4.74
Company 1	9	3.13^*d*^	0.33	3.25	0.55	1.13	2.47	3.49
Company 2	3	3.49	0.46	3.35	0.61	1.07	3.11	4.00
Company 3	3	2.91	1.58	3.44	0.46	1.85	1.14	4.16
Company 4	3	4.55^*d*^	0.16	4.49	0.72	1.04	4.43	4.74
TLR2 (absorbance)								
Overall	18	0.68	0.31	0.59	0.61	1.64	0.15	1.35
Company 1	9	0.85	0.33	0.75	0.80	1.50	0.44	1.35
Company 2	3	0.60	0.01	0.60	0.60	1.02	0.59	0.61
Company 3	3	0.55	0.12	0.51	0.54	1.23	0.46	0.68
Company 4	3	0.37	0.19	0.43	0.32	1.94	0.15	0.51
TLR4 (absorbance)								
Overall	18	0.56	0.21	0.52	0.52	1.52	0.17	0.94
Company 1	9	0.70	0.18	0.70	0.68	1.30	0.45	0.94
Company 2	3	0.48	0.07	0.44	0.47	1.16	0.42	0.56
Company 3	3	0.47	0.12	0.43	0.47	1.27	0.39	0.61
Company 4	3	0.33	0.16	0.32	0.30	1.71	0.17	0.49

a *P* = 0.009 (all *P* values determined by Kruskal-Wallis followed by Wilcoxon rank sum test).

b *P* = 0.009.

c *P* = 0.0002.

d *P* = 0.03.

e AM, arithmetic mean; GM, geometric mean; GSD, geometric standard deviation; LOD, limit of detection.

### TLR activation by the dust samples.

All samples induced significant activation of TLR2 and TLR4 compared to parental HEK reference cells and negative controls (see Fig. S1 in the supplemental material). Three samples with dust levels above 5 mg m^−3^ induced stronger activation of both receptors than positive controls of 10 μg mL^−3^ of lipoteichoic acid (LTA) and 10 ng mL^−3^ of lipopolysaccharide (LPS), respectively. The highest activation values were observed in the dust samples from company 1, and the lowest activations in the dust samples were from company 4. Activation of TLR2 correlated strongly with the dust levels (*r = *0.8, *P* = 0.0001) but weakly with endotoxins (*r* = 0.42, *P* = 0.08) and fungal spores (*r = *0.41, *P* = 0.09). Similarly, activation of TLR4 correlated strongly with dust (*r = *0.71, *P* = 0.001) and moderately with endotoxins (*r = *0.52, *P* = 0.03) and with fungal spore counts (*r = *0.53, *P* = 0.02). This was also supported by linear regression analysis (Table S2). Furthermore, secreted embryonic alkaline phosphatase (SEAP) levels by TLR2 activation were higher than those by TLR4 activation, with the exception of 2 samples (S1 [company 1] and S11 [company 3]), indicating the presence of more ligands for TLR2 than TLR4 in the collected samples.

### Inhalable mycobiome of waste management.

**(i) Fungal diversity patterns.** We found significantly higher operational taxonomic unit (OTU) richness among workers in company 4 (AM ± standard deviation [SD], 557 ± 23) versus companies 1 (276 ± 41), 2 (303 ± 107), and 3 (270 ± 122) (Fig. S2a). In particular, the sample with the highest richness came from a worker who performed a sorting task in company 4. The lowest diversity was measured among workers in company 3, and the sample came from a worker who worked with shredders and presses. Notably, the two samples with the highest and lowest richness were collected from companies in the Oslo region in September. Consistent with the richness patterns, the Shannon diversity index and the evenness were higher in samples from workers at company 4 compared with company 1, but the differences were not significant (*P* = 0.03 and *P = *0.10, respectively) (Fig. S2b and c). The abundance of the 10 most common OTUs in the data set was highest in companies 1 and 3 and lowest in company 4 (Fig. S2d). However, companies 1 (*n* = 432) and 4 (*n* = 447) had a higher number of OTUs than companies 2 (*n* = 94) and 3 (*n* = 195), and a total of 271 OTUs were common to all companies (Fig. S3).

We found no significant linear relationships between fungal diversity measures (i.e., richness, Shannon diversity index, and evenness index) and exposure variables (dust, endotoxin, fungal spores, and actinobacteria) (data not shown). However, we observed that fungal richness was negatively associated with the activation of TLR4 (adjusted *R*^2^ [*R*^2^ adj.] = 0.28, *P* = 0.014) and weakly with TLR2 (*R*^2^ adj. = 0.16, *P* = 0.06) (Fig. S4).

**FIG 2:**
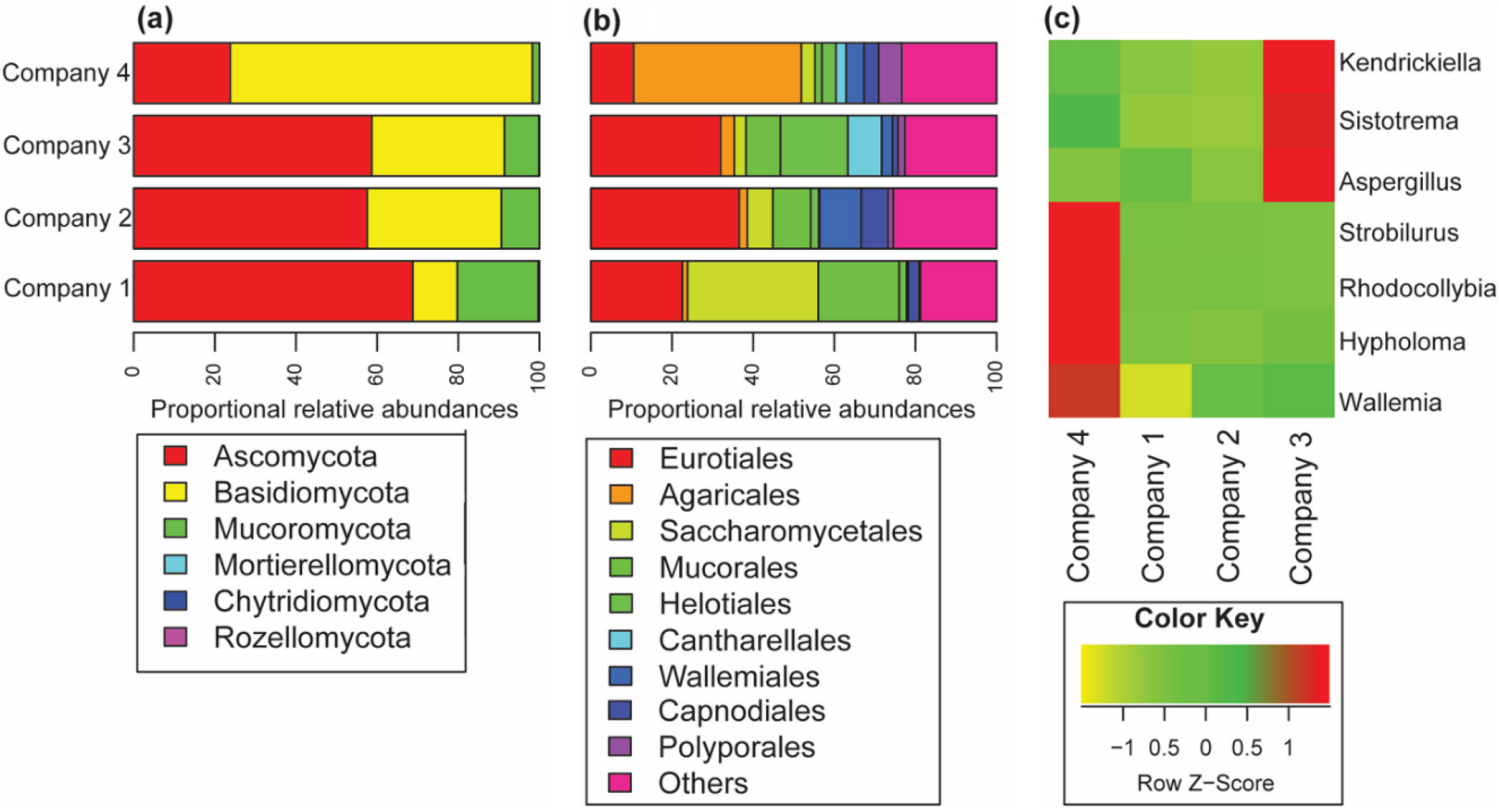
Taxonomic distribution of the fungal communities detected from four recycling plants (companies 1 to 4). (a and b) Proportional relative abundances of airborne fungal compositions at phyla (a) and order (b) levels. The data represent average reads per sample. (c) Hierarchical clustering-based heat plots for proportional abundances of different genera that significantly vary in their abundance among the four companies.

**(ii) Fungal composition by companies and relationship with TLR activation.** Overall, the phylum of Ascomycota (55%) dominated the OTU reads compared to Basidiomycota (33%) and Mucoromycota (2%) (Fig. 2; Table S3). By company, Ascomycota represented 69%, 58%, and 59% of reads in companies 1, 2, and 3, respectively, while in company 4, they represented only 24%. The Basidiomycota dominated the OTU measurements in company 4 (75%), while they accounted for about 33% in companies 2 and 3 and only 11% in company 1 (Fig. 2a).

The phylum Mucoromycota accounted for 20%, 9%, 9%, and 2% in companies 1, 2, 3, and 4, respectively. Among the orders detected, we found that Eurotiales represented 23%, 37%, 32%, and 11%, and Mucorales represented 20%, 9%, 9%, and 2% of the reads in samples from companies 1, 2, 3, and 4, respectively. Other orders, such as Agaricales (41%), Saccharomycetales (32%), and Wallemiales (10%), were mainly present in companies 4, 1, and 2, respectively (Fig. 2b). In addition, the distribution of different fungal genera varied between companies (Fig. 2c). The abundance of the genera *Kendrickiella*, *Sistotrema*, and Aspergillus was significantly higher among workers in company 3 than in other companies, while *Strobilurus*, *Rhodocollybia*, and *Hypholoma* were most abundant in company 4. OTUs with similarity to *Wallemia* genera were more common in samples from workers at company 4, followed by companies 1, 2, and 3. The read abundance of genera *Wallemia*, *Strobilurus*, *Rhodocollybia*, and *Hypholoma* was negatively correlated with TLR activation, indicating that an increasing abundance of these genera is associated with a decreasing activation of TLRs. In contrast, the genera *Candida*, *Rhizopus*, *Alternaria*, *Debaryomyces*, *Vishniacozyma*, and *Mucor* were positively correlated with TLR activation (Fig. S5).

**FIG 3:**
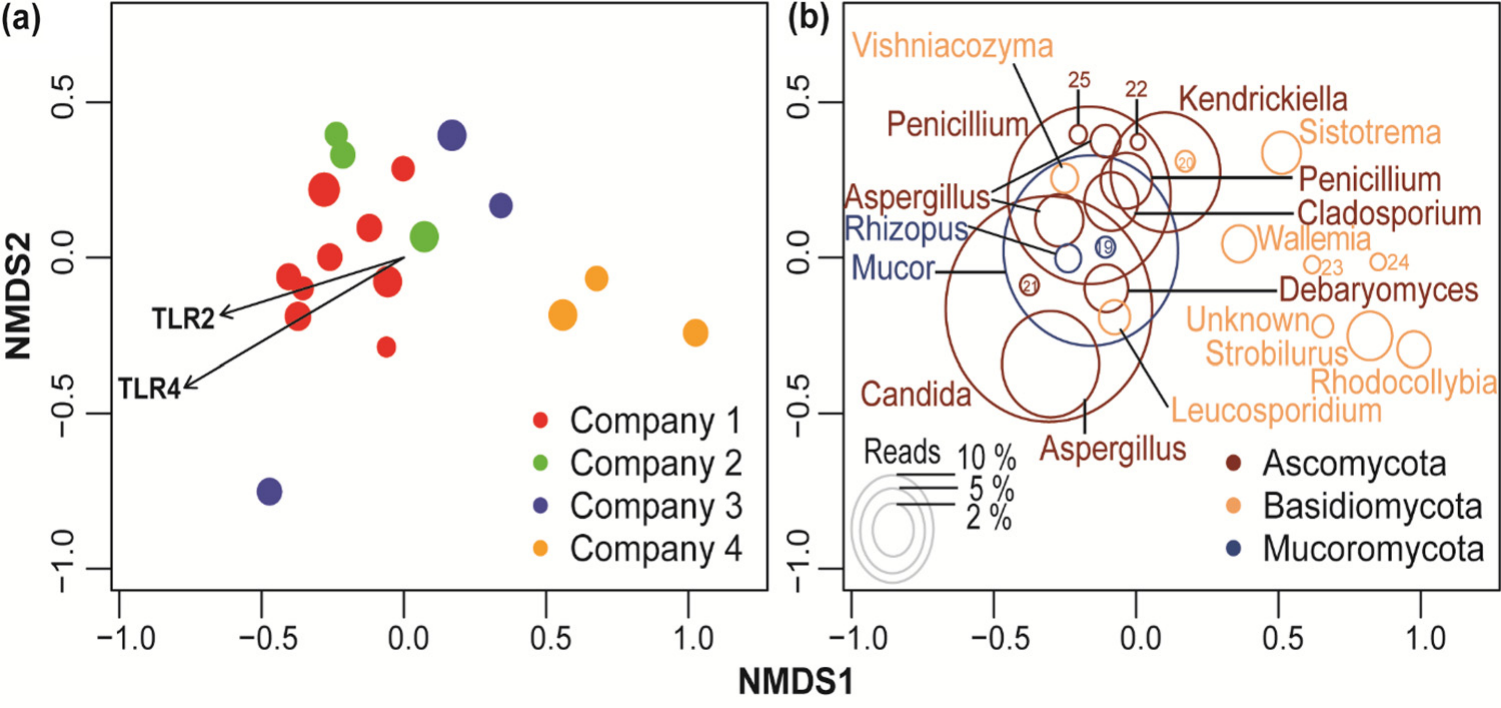
Nonmetric multidimensional scaling (NMDS) ordination analysis for fungal communities detected in personal airborne samples from workers in the four recycling plants. (a) Plot represents samples color coded by companies 1 to 4. Vectors (TLR2 and TLR4 activation variables) shown with the arrow had significant effects (*P < *0.05) on the ordination configuration. (b) Plot showing the fungal OTU composition of four recycling plants. The ordination plot is based on all fungal OTUs present, but only the most common 25 OTUs are shown here, which accounted for 66% of the total reads. The number codes correspond to *Rhizopus* (19), *Melamspora* (20), *Alternaria* (21), *Penicillium* (22), *Cyllindrobasidium* (23), *Hypholoma* (24), and *Pycnopezia* (25).

Multivariate permutational analysis of variance (PERMANOVA) and nonmetric multidimensional scaling (NMDS) analyses (Fig. 3; Table S5) revealed very similar structural patterns and showed that different recycling plants exert a strong impact on the fungal community (*R*^2^ = 0.44; *P* < 0.001). Samples from workers at different companies were well separated, indicating distinct composition, and those from the same company were grouped together in ordination space (Fig. 3a). We found that TLR activation variables (TLR2 and TLR4) significantly correlated with fungal community composition, with both vectors pointing in the same direction as company 1. This indicated that species composition in samples from company 1 induced the highest activation of TLRs, while the species composition in samples from company 4 induced the lowest activation (Fig. 3a). No correlation was found between structural patterns of fungal community and other measured vector variables (dust, endotoxin, fungal spores, actinobacteria).

The distribution of the most abundant fungal OTUs differed between samples from workers at different recycling plants (Fig. 3b; Table S4). In the NMDS ordination space, OTUs matched to the species Candida glaebosa (OTU 1), Debaryomyces hansenii (OTU 10), and Rhizopus arrhizus (OTU 17) showed their optima in the samples from workers in company 1, whereas the species Mucor plumbeus (OTU 2) and Alternaria molesta (OTU 23) showed quite similar distribution in companies 1 and 3, and OTU 3 (Penicillium aethiopicum), OTU 4 (Kendrickiella phycomyces), OTU 5 (Aspergillus fumigatus), and OTU 8 (Aspergillus flavus) showed their highest abundance in company 3. Only OTU 16 (Vishniacozyma victoriae) was more common in company 2, while OTU 6 (Cladosporium delicatulum) and OTU 9 (Strobilurus esculentus) had species optima in company 4 samples. Indeed, S. esculentus and Rhodocollybia buryracea were found almost exclusively in company 4 (99.1% and 99.7% of the reads, respectively), and Sistotrema coronilla and Melampsora epitea were found almost exclusively in company 3 (99.9% and 95.5% of the reads, respectively) (Table S4). The OTU with the highest overall percentage of reads was Candida glaebosa (10.1% of total reads) but was most prevalent in company 1 (87.7% of the reads), while reads in the other companies were 6% or less. Company 3 had a median to high read percentage of most of the top 20 OTUs (Table S4).

## DISCUSSION

In this study, we characterized the occupational exposure to fungi by spores and OTU composition, endotoxins, and actinobacteria present in airborne dust from waste sorting plants. The dust’s potential to induce NF-κB through the activation of TLR2 and TLR4 was also assessed.

The overall dust exposure measured in this study (range, 0 to 50.2 mg m^−3^) is consistent with the concentrations measured in the waste sector in South Africa (35) and Poland (36) and the exposure levels reviewed by Pearson and colleagues (0.1 to 56.14 mg m^−3^) in reference 37. Since airway inflammation symptoms and lung function decline have been associated with even lower dust concentrations (0.1 to 2.1 mg m^−3^) in workers at Norwegian waste recycling plants (2, 3, 38), exposure levels in the present study may be of concern. The dust exposure levels were higher than those measured in Dutch waste management plants (<0.2 to 9.1 mg m^−3^) (4, 39), as well as in Great Britain (0.07 to 17.73 mg m^−3^) (40), South Korea (0.05 to 4.51 mg m^−3^) (41), and in a newly built Norwegian waste treatment plant (0.51 to 18.93 mg m^−3^) (24). Three of the 18 workers were exposed to dust levels higher than the occupational exposure limit for total organic dust in Norway of 5 mg m^−3^ (42). The relatively high dust levels could be attributed to the fact that higher volume and different types of waste materials are recycled today than in the past. Storage of the waste materials prior to processing is likely to promote the growth of sporulating bacteria and fungi, which contribute to airborne organic dust mass (43). Moreover, we suspect an increased emission of airborne particles during the loading and shredding processes, which was supported by high exposure levels measured at the reception and inspection operator position (50.22 mg m^−3^), at the machine operator station (48.55 mg m^−3^), and on the machine driving post (11.04 mg m^−3^). These three workers belong to company 1, which had the highest dust levels compared to the other companies. As tasks with similar dust-generating activities exist in other companies (companies 2, 3, and 4), these high levels may be attributed to higher volumes and/or types of waste materials processed. Similar findings between plants have been previously reported (44), but our results need to be interpreted cautiously because of the small size of samples used in this comparison.

We found relatively high exposure to fungal spores (median, 3 × 10^5^ spores m^−3^; range, 0 to 1.5 × 10^6^ spores m^−3^) and which exceeds the suggested OEL of 1 × 10^5^ spores m^−3^ (45). These levels are comparable to exposure ranges previously reported in the waste sector, 0 × 10^6^ to 2 × 10^6^ spores m^−3^ in Norway (2), 0.038 × 10^6^ to 1.03 × 10^6^ spores m^−3^ in Canada (46), and 0.143 × 10^6^ to 1.65 × 10^6^ spores m^−3^ in Poland (25). On the other hand, much lower exposure levels have been measured in samples from Danish waste transporters (about 33 times the median level) (30) and in Brazilian waste sorting plants (3.2 × 10^4^ spores m^−3^) (5). Higher personal exposure ranges of 0.02 × 10^6^ to 110 × 10^6^, 93 × 10^6^ to 1,140 × 10^6^, and 0.39 × 10^6^ to 5.01 × 10^6^ spores m^−3^ have been measured in waste treatment plants in Germany (47), Denmark (13), and Norway (24), respectively. Of note, we assume that 1 CFU m^−3^ equals 10 spores m^−3^ (48), and this equivalence is an oversimplification to allow for comparisons.

Endotoxins were detected in all samples. The levels ranged from 19 to 5,321 EU m^−3^ and matched previous measurements from Dutch (2 to 1,900 EU m^−3^) (32, 39), British (0.8 to 22656 EU m^−3^) (40), and in Norwegian (223 to 5,277 EU m^−3^) (24) waste treatment plants. Relatively lower endotoxin levels were reported in waste collectors (median, 13 EU m^−3^; range, 4 to 183 EU m^−3^) and in composting workers (median, 3 EU m^−3^; range, 0 to 730 EU m^−3^) in Norway (2, 38). Conversely, higher average concentrations of endotoxins (1,123 EU m^−3^; range, 4 to 6,870 EU m^−3^) were reported from municipal waste treatment facilities in South Korea (41).

Actinobacteria were also detected, but only in samples from company 1, and they ranged from 4 × 10^3^ to 556 × 10^3^ actinobacteria m^−3^. These values are comparable to previous data from Norwegian waste sectors (2) but lower than the exposure levels measured in German composting plants (47).

The fungal sequence data revealed a mycobiome that included a wide spectrum of taxonomic orders belonging to the phyla Ascomycota (Eurotiales, Saccharomycetales, Helotiales, Hypocreales, and Pleosporales), Basidiomycota (Wallemiales, Agaricales, Cantharellales, Capnodiales, Tremellales, Polyporales, and Pucciniales), and Mucoromycota (Mucorales). These mycobiome data are similar to those characterized in the French waste recycling sector (8). The 20 most abundant OTUs (see Table S4 in the supplemental material) matched species belonging to the genera *Candida* (*Debaryomyces*), Aspergillus, *Penicillium*, *Kendrickiella*, *Cladosporium*, *Rhizopus*, *Strobilurus*, *Debaryomyces*, *Wallemia*, *Rhodocollybia*, *Vishniacozyma*, and *Alternaria*. We identified opportunistic pathogens and mycotoxin producers like A. fumigatus and A. flavus and allergenic species such as *Penicillium*, *Cladosporium*, *Alternaria*, and *Wallemia* spp. (49). The percentage of reads showed that *Candida* (including C. glaebosa [teleomorph, Citermyces matritensis] and Debaryomyces hansenii [anamorph, Candida famata]) was the most prevalent genus (12.2%). C. glaebosa and D. hansenii are cryotolerant and halophile (salinity up to 24%) yeasts of marine origin, commonly found in sausage and dairy products (50–52). Their presence in samples from all companies indicates probable contamination of the waste materials with food waste from the dairy or sausage industry. Clinically, C. glaebosa and C. famata have been reported as newly emerging opportunistic pathogens and have been associated with invasive candidiasis (0.2 to 2%) in immunocompromised individuals (53, 54). Aspergillus was the second most common genus and includes A. fumigatus and A. flavus. These two species belong to biosafety level 2, i.e., they can cause opportunistic infections in people with compromised immune systems (55). They are also known as causative agents of aspergillosis and producers of aflatoxin (A. flavus) and gliotoxin (A. fumigatus) (56). The *Rhizopus* genus includes species like Rhizopus microsporus and R. arrhizus (R. oryzae) that belong to the biosafety level 2 group and are classified as facultative causative agents of mucormycosis (57). Wallemia muriae is associated with farmer lungs and bronchial asthma in farmers (58), while Vishniacozyma victoriae is associated with asthma in dwellings (59).

The fungal diversity in the dust samples from the waste sorting plants varied widely between companies and, to some extent, reflected the potential source of waste processed during our sampling campaign. For example, Strobilurus esculentus is widespread on the branches and trunks of conifers, while Rhodocollybia butyraceae, a mushroom, is native to forest floors, where it is common. Another example is Melamspora epitea, a plant leaf parasite (rust fungi). These three species were specifically characterized in samples collected at company 4 (>95% of the reads for these species) and were indicative of the types of materials that company 4 primarily processed during the sampling campaign.

A divergent relationship between fungal species abundance and activation of TLR2 and TLR4 was observed. Increasing abundances of *Candida*, *Debaryomyces*, *Mucor*, *Rhizopus*, *Vishniacozyma*, and *Alternaria* were associated with increased activation of TLR2 and TLR4, while increasing abundances of *Penicillium*, *Kendrickiella*, *Sistotrema*, *Cladosporium*, *Wallemia*, *Strobilurus*, *Leucosporidium*, *Rhodocollybia*, *Hypholoma*, Aspergillus, and *Melamspora* induced decreasing activation of TLR2 and TLR4 (Fig. S5). Furthermore, the overall fungal community composition, measured as OTU richness, was negatively correlated with TLR4 activation (Fig. S4) in contrast to the fungal spore counts, which were positively correlated with TLR2 and TLR4 (Table S2). To the best of our knowledge, this is the first study to describe such a divergent relationship between biological response in an *in vitro* cell system with fungal exposure in a waste sorting environment. A possible explanation of the negative association could be related to the specificity and affinity of TLR4 for *O*-linked mannans/glycoproteins of fungal spore wall components (60). Because the distribution and the cell wall concentrations of these components can vary between species (61), it is likely that the increasing prevalence of species with reduced concentrations or masked *O*-glycoproteins may contribute to a decrease in TLR4 activation. In particular, *Candida* yeasts have glycoproteins exposed directly on the cell wall surface, in contrast to Aspergillus spores whose glycoprotein layer is masked by melanin and hydrophobin layers. For the spore numbers, the positive correlation with TLR2 and TLR4 could be explained by the dominance of spore production by a few sporulating fungi. Thus, increasing spore levels, but only from a few species, will increase levels of phosphor-lipomannan, which activates TLR2 (62), and *O*-linked glycoproteins, which activate TLR4 (60). Spore levels did not correlate with OTU richness, Shannon index, or evenness (data not shown) but showed a negative trend relationship comparable to data reported on fungal diversity in the sawmills (63). We can also draw a parallel between these findings and the results from Viegas and colleagues, who found negative-trend relationships between inflammatory cytokine levels and bacterial and fungal diversity in dust collected in the Portuguese waste recycling sector (9), considering that the production of inflammatory cytokines is, to some extent, intrinsically linked to NF-κB induction through TLR activation. The underlying mechanisms of this negative relationship remain unclear; however, we can speculate on the proinflammatory properties of large insoluble beta-glucan components versus the anti-inflammatory effects of soluble beta-glycans (64). As such, increasing levels of soluble beta-glycans are likely linked to increasing abundance for fungal species.

Compared to previously reported levels of bioaerosols in waste management, our results indicate moderate to high exposure levels of dust, endotoxins, and fungal spores, all of which moderately to weakly correlate with NF-κB-induced SEAP production through TLR2 and TLR4 activation. We found a strong correlation between total dust levels and TLR2 and TLR4 activation, suggesting that dust particles carry many ligands and/or agonists for these receptors. Although both TLR2 and TLR4 were activated by the dust, the NF-κB-induced response was highest with TLR2 and in most samples (16 out of 18) (Fig. S2). This indicates that the dust contained more TLR2 ligands to which chronic exposure may shape the adaptive immune response toward Th2 and regular T cell (Treg) differentiation pathways characterized by allergic immune responses (65, 66). Activation of TLR4 indicates the presence of ligands capable of promoting an adaptive immune response toward Th1 and Th17 differentiation, especially under chronic exposure-related activation (67).

The endotoxin exposure levels were mainly in the range of concentrations that can cause inflammation (100 EU m^−3^), systemic effects (1,000 EU m^−3^), or toxic pneumonitis (2,000 EU m^−3^) (40). Even relatively low exposure levels in waste workers have been shown to be positively associated with inflammatory markers such as myeloperoxidase (2, 38). The correlation between TLR4 activation and the endotoxin levels was surprisingly weak, albeit significant (*R*^2^ adj. = 0.20; *P* = 0.03). The presence of different types of endotoxins with different levels of acylation of lipid A may explain this. Low acylated lipid A forms of endotoxins, in contrast to hexa-acylated forms, are poor stimulators of the TLR4-MD-2 complex (68).

The initial activation of PRRs by pathogen-associated molecular patterns (PAMPs) present in the dust, followed by induction of immunological responses, plays a fundamental role in the development of adaptive inflammatory responses. Chronic exposure with repetitive oxidative and inflammatory reactions is associated with chronic diseases and a decline in lung function over time (26). By characterizing the potential of inhalable dust to induce NF-κB responses through TLR activation as immunological key events that potentially lead to adverse health outcomes, the present study provides a useful biological endpoint approach to consider in risk assessment and future epidemiological studies.

The significant variation in fungal spore levels and fungal species diversity between waste sorting plants suggests that different exposure-related health effects can be expected among waste company employees. Therefore, fungal exposure assessments, both by the company as well as by tasks, can be crucial for evaluating health risks. Compared to the extended characterization of suspected dust components, this is an improvement in exposure and hazard characterization toward parameters that are mechanistically closer to potential health effects. Further epidemiological studies linking key immunological events, as reported here, and quantified biomarkers of adverse health outcomes in workers are needed to confirm such an association. Despite the new insights into exposure and hazard characterization from *in vitro* activation of TLR2 and TLR4, the small sample number in this study is a limitation. Further studies with a larger exposure sample size using TLR activation by dust from the waste management sector are needed for more definitive conclusions.

### Conclusions.

Besides the exposure characterization to dust, fungal spores, OTU diversity, endotoxins, and actinobacteria in Norwegian waste recycling plants, the present study provided new insights into the immunological key mechanisms related to fungal exposure. Overall, workers were exposed to potentially hazardous levels of dust, endotoxins, and fungal spores. A multifactor-associated NF-κB-induced response through the activation of TLR2 and TLR4 by the collected dust provided a measure of the potential immunological risk effect by considering all potential hazardous contents, including the fungal spore levels and diversity, as well as endotoxins, bacteria, and other uncharacterized components. The positive correlation of NF-κB-induced cell signaling to fungal spore levels in contrast to the negative correlation with species diversity suggests that fungal spore levels as such contribute only partially to the key immunological outcomes and that identification at the species level is crucial to assessing exposure-related health effects.

Future hazard characterization studies in waste management and other similar environments need to focus on the role of the complex composition of dust and associated health effects on workers to gain a more holistic view of the exposure-response relationships leading to disease.

## MATERIALS AND METHODS

### Study sites and sample collection.

**FIG 4:**
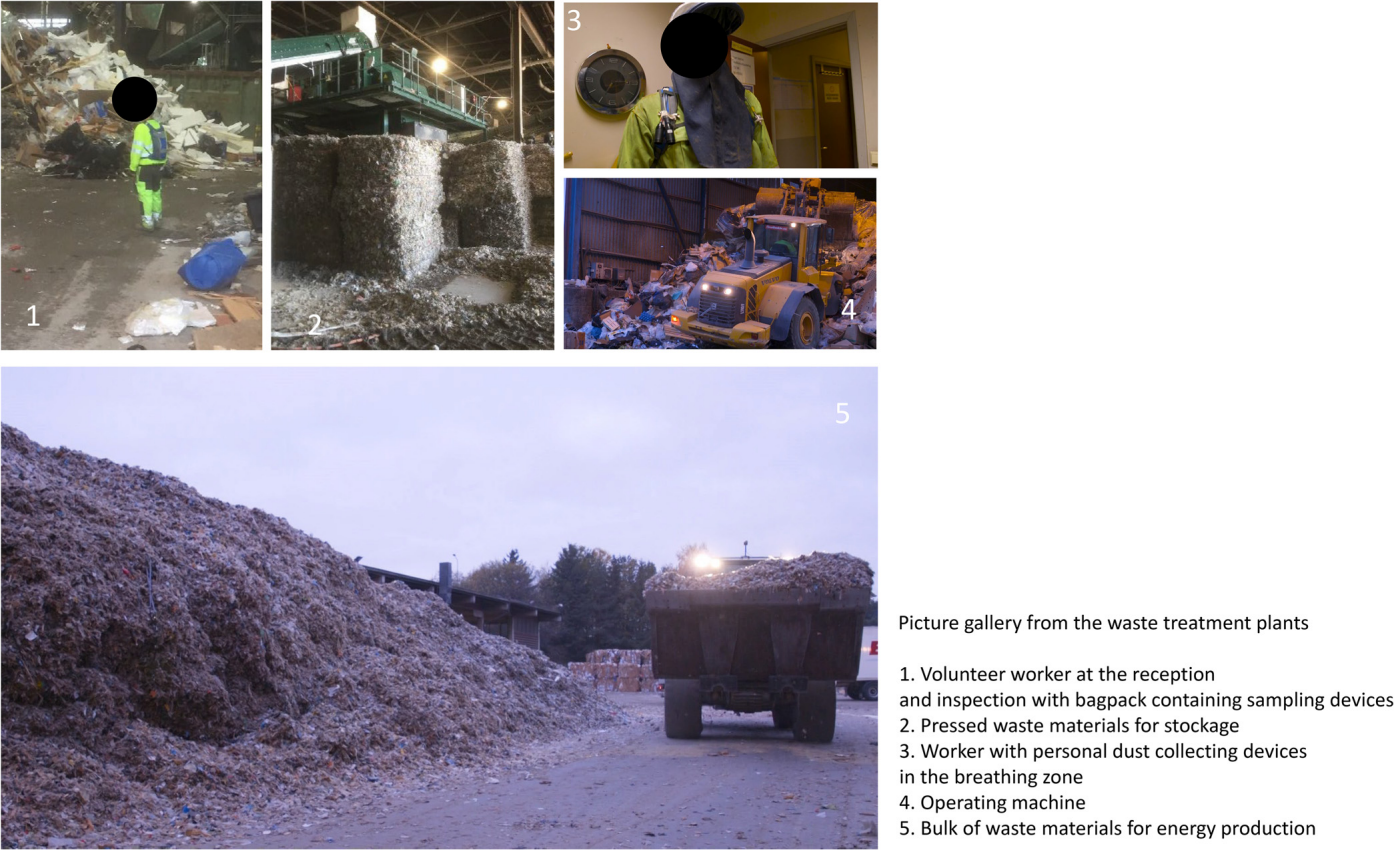
Picture gallery from the waste treatment plants. (1) Volunteer worker at the reception and inspection with bag pack containing sampling devices. (2) Pressed waste materials for stockage. (3) Worker with personal dust-collecting devices in the breathing zone. (4) Operating machine. (5) Bulk of waste materials for energy production.

Air samples were collected at four waste recycling plants in Norway. The recycling plants processed a wide range of waste, but mainly waste from offices and various industries. Different types of waste, such as cardboard, paper, plastics, gypsum board, old windows, fillers, and garden materials, are generally collected and handled through different sorting lines (Fig. 4). Wooden waste materials like furniture are ground/shredded, the metal parts are sorted automatically, and the remaining wooden fraction is processed and used in energy production. Organic waste or materials contaminated by food waste were also delivered to the waste collection point, but not processed in the plants. These were instead loaded onto trucks after inspection and transported to other sites for incineration. The following work tasks, which are probably most exposed, were selected for the measurements: (i) waste reception and inspection, (ii) material sorting line, (iii) material shredding and pressing, (iv) machine operator/driving, and (v) storage. All employees in reception and control, sorting, shredding, and pressing, as well as machine operators, took part in the sampling campaign. The workers were organized into two 8-h shifts, with the heaviest workload on the morning shift. Morning full-shift personal air samples from 18 workers were therefore collected in August, September, and October 2017 at all 4 plants. Detailed sample information is summarized in Table S1 in the supplemental material. Of note, ethical approval is not required for occupational exposure assessment in Norway as long as the data collected cannot be linked to individual workers and no personal health information and/or biological materials are gathered from the worker. However, we did have verbal agreements with the workers who volunteered to carry sampling devices.

Personal samples were collected simultaneously with three parallel sampling cassettes worn by each participant. Each worker carried a backpack loaded with pumps attached to each set of filter cassettes placed in the worker's breathing zone. One sampling set was used for quantification of total dust, fungal spores, and actinobacteria levels, and a second set was used for metabarcoding. Dust samples were collected by impaction on preconditioned and preweighted 25-mm hydrophilic polycarbonate membrane filters with a pore size of 0.8 μm (Merck KGaA, Darmstadt, Germany) mounted in antistatic polypropylene total dust filter cassettes (Pall Laboratories, Port Washington, NY, USA). A third sampling set with 25-mm glass fiber filters (1 m, GF/A, Whatman, UK) mounted in personal air sampler cassettes (PAS-6; 69) was used for the collection of endotoxins. Air pumps (GSA5200; GSA Messgerätebau GmbH, Ratingen, Germany) were operated at an average flow rate of 2.0 (±10%) L per minute. The total sampling time varied between 3.9 and 7 h (mean, 5.7 h).

### Gravimetric analysis of the dust.

The total dust content was determined gravimetrically using a microbalance (MC210p; Sartorius AG, Göttingen, Germany). The sample-loaded filters were acclimatized (temperature of 20 ± 1°C and relative humidity of 40% ± 2%) for 48 h before being subjected to gravimetric analysis. Two unexposed blank field filters were included, and the average weight was used as a blank to adjust the final dust weight. The detection limit corresponded to 0.02 mg/filter.

### Dust elution from filter membrane.

The filter samples were eluted in 5 mL phosphate-buffered saline (PBS) with 0.1% bovine serum albumin (BSA) in 15-mL centrifuge tubes and subjected to 5 min sonication followed by orbital shaking at room temperature for 60 min. The dust suspension was then transferred to a new tube, and the process was repeated once with 2 mL PBS with 0.1% BSA for 25 min. The final dust suspension was aliquoted and kept at −20°C until microscopic analysis and *in vitro* experiments.

### Field emission scanning electron microscopy analysis of actinobacteria and fungal spores.

For microscopic analysis, 0.5 mL of each aliquoted sample was filtered onto a 25-mm polycarbonate filter (pore size, 0.45 μm; Merck KGaA, Darmstadt, Germany). The filters were then air-dried under sterile conditions and mounted onto 25-mm-diameter aluminum pin stubs (Agar Scientific Ltd., Stansted Essex, UK) using double-sided adhesive carbon discs. The filter samples were coated with 5 to 6 nm platinum in a Cressington 200HR sputter coater (Cressington Scientific Instruments Ltd., Watford, UK). Coated samples were analyzed using a field emission scanning electron microscope (FESEM) as previously described in reference 70. Briefly, the microscope was operated in secondary electron imaging mode with an acceleration voltage of 15 keV, an extraction voltage of 1.8 kV, and a working distance of 10 to 11 mm. Fungal and actinobacteria spores were identified by morphological features and counted in 100 randomly selected imaged fields at ×3,000 magnification. The spore counts were given as the number of spores per cubic meter of air.

### Fungal community characterization.

**(i) DNA extraction and metabarcoding.** DNA was extracted from exposed filters as well as three unexposed controls using the E.Z.N.A. soil DNA kit (Omega Bio-tek, Norcross, GA, USA). The filters were transferred to disruptor tubes, and 800 μL SLX-Mlus buffer was added. The samples were homogenized (2× 1 min at 30 Hz) by a TissueLyser (Qiagen, Hilden, Germany) and stored at −20°C until the next step. The samples were then thawed at 70°C, incubated for 10 min, and then homogenized again using the same procedure. Thereafter, the samples were chilled on ice before adding 600 μL chloroform. The samples were then vortexed and centrifuged at 13,000 rpm for 5 min at room temperature (RT). The aqueous phase was transferred to a new tube with an equal volume of XP1 buffer and vortexed. Samples were transferred to HiBind DNA Mini column and processed by following the manufacturer’s guidelines. The extracted DNA was eluted in 50 μL elution buffer. The fungal internal transcribed spacer 2 (ITS2) region was targeted with the forward primer ITS4 (71) and the reverse primer gITS7 (72) (Tables 2 and 3), with barcodes of 6 to 9 bp. The ITS2 region was selected because of less amplification bias due to length differences and the development of less biased primers. The PCR contained 2 μL DNA template and 23 μL master mix containing 14.6 μL Milli-Q water, 2.5 μL 10× Gold buffer, 0.2 μL deoxynucleoside triphosphate (dNTP) (25 nM), 1.5 μL reverse and forward primers (10 μM), 2.5 μL MgCl_2_ (50 mM), 1.0 μL BSA (20 mg/mL), and 0.2 μL AmpliTaq Gold polymerase (5 U/μL; Applied Biosystems, Thermo Fisher Scientific). Negative PCR controls were included. Amplification was performed by initial denaturation (95°C for 5 min), followed by 32 cycles of denaturation (95°C for 30 s), annealing (55°C for 30 s), and elongation (72°C for 1 min). A final elongation step was included (72°C for 10 min). The PCR products were normalized with the SequalPrep normalization plate kit (Invitrogen, Thermo Fisher Scientific, Waltham, MA, USA) and eluted in 20 μL elution buffer. The resulting PCR products were pooled before concentration and purification by Agencourt AMPure XP magnetic beads (Beckman Coulter, CA, USA). The concentration of the purified pools was determined by Qubit (Invitrogen, Thermo Fisher Scientific, Waltham, MA, USA) before the library was sent to Fasteris SA (Plan-les-Ouates, Switzerland) for barcoding with Illumina adapters, spiking with PhiX, and sequencing with 2 × 250-bp paired-end reads with Illumina MiSeq (Illumina, San Diego, CA, USA).

**TABLE 2:** Primers used in the amplification of the ITS2 region

Primer	Sequence^*a*^	Reference
Forward primer ITS4	5′-xCTCCGCTTATTGATATG	71
Reverse primer gITS7	5′-xGTGARTCATCGARTCTTTG	72

a x, barcode per sample.

**TABLE 3:** Samples used in this study

Sample	Forward barcode per sample	Reverse barcode per sample
S347	NNNGTATGTTC	NNGTATGT
S348	NGTCAATTC	NNNGTCAAT
S349	NNAGCCTCTC	NAGCCTC
S350	NNNTCGTTATC	NNTCGTTA
S351	NTGTGGCTC	NNNTGTGGC
S352	NNNCTCTGCTC	NCTCTGC
S354	NNACAGGTTC	NACAGGT
S355	NNNTCCGCTTC	NNTCCGCT
S356	NGTCCGGTC	NNNGTCCGG
S357	NNCATTAGTC	NCATTAG
S358	NNNGAAGCTTC	NNGAAGCT
S359	NGATATTTC	NNNGATATT
S360	NNNAGCTGGTC	NAGCTGG
S361	NCGCGATTC	NNNCGCGAT
S362	NNACATTGTC	NACATTG
S363	NNNCCAAGGTC	NNCCAAGG
S364	NACCATATC	NNNACCATA
S366	NNNGTCTTATC	NNGTCTTA

**(ii) Bioinformatic workflow.** Forward and reverse sequences were retrieved from Fasteris SA, which we independently demultiplexed using Cutadapt v2.7 (mismatches between barcode tags and sequence primer = 0, minimum sequence length = 100 bp) (73). DADA2 v12 (74) was used to filter low-quality sequences (maximum expected error = 2.5) and to correct errors based on an implemented machine learning model. Then, we merged the error-corrected forward and reverse sequences (minimum overlap = 5 bp). Chimeras were filtered out using the Bimera algorithm in DADA2 (default parameters). The resulting amplicon sequence variants were clustered into operational taxonomic units (OTUs; 97% similarity) using VSEARCH (75) before using LULU (76) to correct for possible OTU oversplitting (default parameters). Taxonomy was assigned to the resulting OTU table by BLAST (77) and the UNITE database (78). All negative controls were automatically removed during bioinformatics due to insufficient sequences. The final data set contained 2,516 OTUs.

### Analysis of endotoxins.

Exposed glass fiber filters were transferred to 15-mL glass tubes and eluted in 5 mL endotoxin-free water containing 0.05% Tween 20 by orbital shaking for 1 h. The filters were then removed from the tube, and the suspension was centrifuged at 1,000 × *g* for 15 min to pellet the particulate fraction. The supernatant underwent a second centrifugation step before being aliquoted without the pellet and stored at −20°C until analysis. For endotoxin quantification, supernatants were diluted 20 to 50 times before a Limulus amoebocyte (LAL) kinetic-QCL assay was applied according to the manufacturer’s instructions (Lonza Ltd., Basel, CH). The detection limit of the assay was estimated at 0.5 to 2.5 EU/filter depending on the dilution rate. Parallel controls consisting of samples spiked with 10 μL endotoxin solution (50 EU mL^−1^) and blank samples were run to assess possible inhibition or enhancement of the sample matrix. The endotoxin concentrations were determined by kinetic measurement of absorbance at 405 nm using a BioTek spectrophotometer (BioTek Instruments Inc., VT, USA) in accordance with the five-point standard curve with concentrations ranging from 0.005 to 50 EU mL^−1^.

### *In vitro* assay of NF-κB response through TLR2 and TLR4 activation.

Human embryonic kidney (HEK) 293 reporter cells for TLR2 and TLR4 (Invivogen, France) were exposed to the eluted dust samples to study their potential to induce NF-κB responses. The reporter cells specifically expressed TLR-inducible reporter genes encoding secreted embryonic alkaline phosphatase (SEAP) via the NF-κB signaling pathway. SEAP is secreted extracellularly and can be quantified in the supernatant after NF-κB induction by TLR activation. The exposure experiments were performed in a 96-well plate following the procedure described in reference 79 with some minor modifications. Briefly, we transferred 180 μL per well of 2.8 × 10^5^ cells mL^−1^ in culture media (Dulbecco’s modified Eagle medium [DMEM] with 10% fetal bovine serum and manufacturer-recommended HEK Blue selection antibiotics) and incubated for 3 h at 37°C (5% CO_2_, high humidity) to allow cells to settle at the bottom and relieve stress. Thereafter, 20 μL of the dust suspension was added in triplicate, and the cells were incubated overnight for 22 h. The following day, 20 μL of cell supernatant was transferred to new plates and supplemented with freshly made 180 μL Quanti-Blue solution (Invivogen, France). After 180 min of incubation, the developed color was measured spectrophotometrically at a wavelength of 649 nm using SpectraMax i3 equipped with SoftMax Pro 6.3.1 software (Molecular Devices LLC, San Jose CA, USA). We included sample wash buffer (PBS plus 0.1% BSA) as a negative control, lipoteichoic acid (LTA; Invivogen, France) as a positive control for TLR2, and ultrapure lipopolysaccharide (LPS; Invivogen, France) as a positive control for TLR4 and Zymosan (Merck KGaA, Darmstadt, Germany) that activate both receptors. Note that a parallel plate with culture media with samples only was included to check absorbance due to dust alone. Final data were presented as arithmetic means of the three replicate measurements adjusted for background from media with samples only.

### Data treatment and statistical analysis.

Prior to statistical analysis, all concentrations equal to zero for fungal spores, actinobacteria, and endotoxins were arbitrarily replaced by the limit of detection (LOD)/√2 (80, 81). The levels of the exposure components were reported as arithmetic mean (AM) with standard deviation and geometric mean (GM) with geometric standard deviation. For comparison between companies, the nonparametric Kruskal-Wallis (K-W) test was used because the data for dust, endotoxins, actinobacteria, and TLR2 were not normally distributed (Shapiro test, *P* ≤ 0.05) and because of the small sample size and unbalanced data set. After a significant K-W-test, *post hoc* Wilcoxon rank sum tests (or Mann-Whitney test) with Bonferroni adjustment (*P* ≤ 0.0125 for 4 companies) were used. Visualization of TLR activation was performed using bar graphs. In addition, we applied correlation and linear regression analysis to show the relationship between different exposure components and TLR activation. For the multiple-comparison tests of exposure agents, a two-sided *O*-value of 0.013 was considered statistically significant. The above statistical analyses were performed using Stata 16 SE (StataCorp LP, College Station, TX, USA).

For fungal diversity, statistical analyses were performed in R v4.0.3 (82). To achieve homogeneity of variances, the absorbance values of the TLR2 and TLR4 activation were transformed to zero skewness and expressed on a 0 to 1 scale before the analyses, and the fungal community matrix (OTU table) was arcsine transformed prior to analyses as used earlier (83). For fungal diversity analyses, the OTU table was normalized by randomly subsampling 10,982 reads per sample, which was the lowest read count for a sample. Richness, Shannon index, and evenness measures were computed using the vegan package, and intercompany differences were examined using ANOVA followed by Tukey’s honestly significant difference (HSD) *post hoc* test of the agricolae package and visualized using violin plots. Linear regression analyses were also performed to examine the relationship between fungal abundance and TLR2/TLR4 activation. We also calculated Pearson’s correlations between fungal genera abundance and TLR4 as well as TLR2 activation absorbances using the package corrplot. The variation in the abundance of fungal genera from different companies was tested using ANOVA (Benjamini-Hochberg false-discovery rate [FDR] correction was applied) followed by a Tukey’s HSD *post hoc* test and illustrated by heat plots based on hierarchical clustering. Species accumulation curves for the number of cumulative OTUs for different companies were calculated using the function specaccum from the vegan package (84) with 10,000 permutations. The overall taxonomic composition of fungi at the phylum level and order level among companies was visualized using stacked bar charts. The Bray-Curtis dissimilarity index was used to generate community distance matrices and further used in all multivariate analyses. To examine the relative importance of different factors (companies) and vectors (TLR2 and TLR4 activation), variables on fungal community structural patterns, the multivariate permutational analysis of variance (PERMANOVA) test was used, as implemented in the Adonis function of the package vegan. The PERMANOVA analysis was performed using a forward selection practice to improve the final model. We first examined single-variable models and then included significant factors in the final model in order of their *R*^2^ values. In addition, global nonmetric multidimensional scaling (GNMDS) ordination analysis was used to visualize the effects of studied variables on the composition of fungal community using the metaMDS function of the vegan package. Vector variables and centroids of the factor variables were fitted to GNMDS plots using the envfit function, and the ordiellipse function was used to plot the 95% confidence intervals (CIs) of the different companies.

### Data availability.

All sequence data and related information are available at NCBI with BioProject accession no. PRJNA925793.
