# Supplementary material for: Divergent TLR2 and TLR4 Activation by Fungal Spores and Species Diversity in Dust from Waste Sorting Plants
Source: Appl Environ Microbiol. 2023 Mar 1;89(3):e01734-22. doi: 10.1128/aem.01734-22 (PMC10056968; doi:10.1128/aem.01734-22)
Supplement: Supplemental file 1 — Supplemental material. Download aem.01734-22-s0001.pdf, PDF file, 1.0 MB [file aem.01734-22-s0001.pdf]

Supplementary information for:

"Divergent TLR2 and TLR4 activation by fungal spores and species diversity in dust from waste sorting plants"

Short tittle: ***"Immune responses of dust from waste sorting plants"***

Anani K Afanou[1]; Sunil Mundra [2,3]; Eva Lena Fjeld Estensmo[4]; Ine Pedersen[1]; Jens Rasmus Liland[1]; Elke Eriksen[1]; Pål Graff[1]; Tonje Trulssen Hildre[5]; Karl-Christian Nordby[1]; Anne Straumfors[1]

[1]National Institute of Occupational Health (STAMI), P.O. Box 5330, 0304, Oslo, Norway

[2]Department of Biology, College of Science, United Arab Emirates University, Al-Ain, Abu-Dhabi, UAE

[3]Khalifa Center for Genetic Engineering and Biotechnology, United Arab Emirates University, Al Ain, 15551, United Arab Emirates

[4]Univeristy of Oslo, Department of Bioscience, P.O. Box 1066 Blindern, 0316 Oslo, Norway

[5]Oslo University Hospital, Department of Lung Medicine, Oslo Norway

Table S**1:** Details on sampling

Company	Region	Sampling dates	Ambient temperature (°C)		Ambient humidity (RH%)		Waste materials processed	Tasks of worker	Number of workers	Number of samples for dust analysis	Number of samples for endotoxin analysis	Number of samples for metabarcoding
			min	max	min	max						
#1	Eastern	21.08.2017	10.6	20.4	-	-	Construction waste materials (fillers, metals, cement…), plastics, paper carboards, wooden materials and dangerous wastes	Reception & inspection	2	2	2	2
								Machine operator/driving	1	1	1	1
#1	Eastern	22.08.2017	12.2	18.6	-	-		Reception & inspection	2	2	2	2
								Machine operator/driving	1	1	1	1
#1	Eastern	10.10.2017	1.2	9.9	-	-		Machine operator/driving	3	3	3	3
#2	Eastern	30.08.2017	10.8	18	-	-		Reception & inspection	1	1	1	1
								Ramp chair operators	2	2	2	2
#3	Western	01.09.2027	10.2	13.1	66	98		Reception & inspection	1	1	1	1
								Sorting	1	1	1	1
								Shredding & pressing	1	1	1	1
#4	Eastern	29.09.2027	10.4	11.4	77	83	Construction waste materials (fillers, metals, cement…), plastics, glass, paper carboard, wooden materials, garden waste materials	Sorting	2	2	2	2
								Machine operator/driving	1	1	1	1
								Total	18	18	18	18

Table S2: Linear regression analysis

VARIABLES	TLR2	TLR2	TLR2	TLR2	TLR4	TLR4	TLR4	TLR4
Dust	**0.597*** **				**0.616*** **			
	(0.122)				(0.144)			
Fungal_spores		**0.362** **				**0.464** **		
		(0.163)				(0.168)		
Actinobacteria			-0.00126				0.148	
			(0.260)				(0.282)	
Endotoxin				**0.401***				**0.482** **
				(0.196)				(0.208)
Constant	0.440***	0.387***	0.558***	0.356***	0.437***	0.340***	0.476**	0.317**
	(0.0440)	(0.0921)	(0.157)	(0.111)	(0.0521)	(0.0949)	(0.171)	(0.118)
F	24.01	4.94	0.00	4.22	18.3	7.63	0.27	5.36
**R^2 adjusted**	**0.58**	**0.19**	-0.06	**0.16**	**0.50**	**0.28**	-0.045	**0.20**
p-values model	0.0002	0.04	0.99	0.06	0.0006	0.014	0.6	0.03
Observations	18	18	18	18	18	18	18	18

Standard errors in parentheses *** p<0.01, ** p<0.05, * p<0.1

NB: all data were normalized before the regression analysis and the regression model is as followed:

TLRs = Constant + Exposure agents + Residuals

Where:
TLRs represent TLR2 or TLR4
Exposure agents represent dust, fungal spores, endotoxins or actinobacteria

Table S3: Taxonomic composition of fungal communities recovered from different recycling companies (1-4). Mean abundance (%) and mean occurrences (%) are shown for overall fungal dataset as well as for each company.

	**Total (%)**		Company 1 (%)		Company 2 (%)		Company 3 (%)		Company 4 (%)	
Taxonomy	**Reads**	**Occurrence**	Reads	**Occurrence**	Reads	**Occurrence**	Reads	**Occurrence**	Reads	**Occurrence**
**Ascomycota**	**54.7**	**39.4**	68.8	47.3	57.5	40.7	58.7	45.3	23.8	23.7
Eurotiales	**23.3**	**5.7**	22.5	6.4	36.6	7.5	32.0	6.2	10.5	3.6
Saccharomycetales	**15.6**	**6.4**	32.2	9.1	6.3	6.6	2.9	5.5	3.4	2.7
Helotiales	**6.4**	**3.0**	1.8	2.7	1.9	2.3	16.6	5.0	3.4	2.9
Capnodiales	**2.7**	**2.0**	2.7	2.1	6.7	1.8	1.3	2.1	3.5	1.7
Pleosporales	**1.8**	**3.5**	2.5	4.0	3.2	4.2	1.3	3.2	0.7	2.4
Hypocreales	**1.4**	**4.3**	2.8	5.5	0.4	4.1	0.5	5.4	0.1	1.9
**Basidiomycota**	**33.3**	**57.7**	11.0	48.3	33.1	56.7	32.7	51.9	74.5	75.3
Agaricales	**11.2**	**15.6**	1.3	10.7	2.0	13.3	3.3	10.2	41.4	26.6
Cantharellales	**3.0**	**4.0**	0.2	2.7	0.3	2.8	8.4	4.2	2.5	6.3
Wallemiales	**2.5**	**0.8**	0.2	1.0	10.2	1.1	2.7	0.7	4.5	0.5
Leucosporidiales	**2.1**	**0.7**	2.6	0.7	0.4	1.0	0.3	0.4	3.9	0.6
Polyporales	**2.1**	**6.9**	0.3	5.4	1.3	6.0	1.7	6.2	5.8	10.0
Tremellales	**1.7**	**2.6**	1.8	2.8	9.0	3.7	0.7	2.9	0.8	1.7
Sporidiobolales	**1.6**	**2.4**	1.2	2.6	2.3	3.1	2.4	2.5	1.3	1.8
Pucciniales	**1.5**	**0.6**	0.3	0.7	0.3	1.1	3.8	0.6	1.0	0.4
**Mucoromycota**	**12.0**	**2.8**	20.3	4.2	9.4	2.4	8.9	2.8	1.7	1.0
Mucorales	**11.8**	**2.1**	20.0	3.0	9.4	2.1	8.5	2.0	1.7	0.8
**Chytridiomycota**	**0.0**	**0.1**	0.0	0.2	0.0	0.2	0.0	0.1	0.0	0.0
**Rozellomycota**	**0.0**	**0.0**	0.0	0.0	0.0	0.0	0.0	0.2	0.0	0.0

Table S4: Taxonomic affinity, read abundance and occurrences of the 20 most abundant operational taxonomic units (OTUs) detected in four recycling plants (company 1 – 4). For overall communities both occurrences and % total reads are shown, but for individual companies only total reads (%) data is provided.

			Total		Total reads (%) by company			
**OTU_ID**	**Top hit**	**Taxonomic rank**	**Occurances**	**Reads (%)**	**Company 1**	**Company 2**	**Company 3**	**Company 4**
OTU1	Candida_glaebosa_SH1516576.08FU	Saccharomycetales (A)	18	10.1	**87.6**	2.4	6.0	4.0
OTU2	Mucor_plumbeus_SH2129993.08FU	Mucorales (M)	18	8.6	**46.5**	10.1	**36.5**	7.0
OTU3	Penicillium_aethiopicum_SH2190001.08FU	Eurotiales (A)	18	8.0	21.2	20.5	**40.6**	17.7
OTU4	Kendrickiella_phycomyces_SH1517387.08FU	Helotiales (A)	18	5.4	3.3	1.9	**81.1**	13.6
OTU5	Aspergillus_fumigatus_SH1162325.08FU	Eurotiales (A)	18	4.8	12.1	1.7	**77.0**	9.2
OTU6	Cladosporium_delicatulum_SH2320203.08FU	Capnodiales (A)	18	2.6	19.2	21.9	18.1	**40.8**
OTU8	Aspergillus_flavus_SH1161185.08FU	Eurotiales (A)	18	2.4	31.7	6.7	**57.0**	4.6
OTU9	Strobilurus_esculentus_SH1880178.08FU	Agaricales (B)	15	2.2	0.6	0.2	0.1	**99.1**
OTU10	Debaryomyces_hansenii_SH2154634.08FU	Saccharomycetales (A)	18	2.1	**40.1**	10.3	19.5	30.1
OTU11	Sistotrema_coronilla_SH1506094.08FU	Cantharellales (B)	4	1.9	0.0	0.0	**99.9**	0.1
OTU12	Wallemia_muriae_SH1605561.08FU	Wallemiales (B)	18	1.7	0.9	18.5	**33.4**	**47.2**
OTU13	Rhodocollybia_butyracea_SH1646573.08FU	Agaricales (B)	10	1.6	0.1	0.3	0.0	**99.7**
OTU14	Leucosporidium_creatinivorum_SH1651363.08FU	Leucosporidiales (B)	18	1.5	**45.7**	2.1	9.7	**42.5**
OTU15	Aspergillus_appendiculatus_SH2321620.08FU	Eurotiales (A)	18	1.5	19.3	23.9	**37.9**	19.0
OTU16	Vishniacozyma_victoriae_SH1528207.08FU	Tremellales (B)	18	1.3	28.0	**43.0**	17.4	11.6
OTU17	Rhizopus_arrhizus_SH1510152.08FU	Mucorales (M)	16	1.3	**80.4**	13.0	3.0	3.6
OTU21	Rhizopus_microsporus_SH2377989.08FU	Mucorales (M)	17	0.9	19.6	1.2	**77.6**	1.6
OTU22	Melampsora_epitea_SH1572516.08FU	Pucciniales (B)	15	0.9	2.7	0.5	**95.5**	1.3
OTU23	Alternaria_molesta_SH2127294.08FU	Pleosporales (A)	18	0.9	**53.3**	3.2	**40.7**	2.8
OTU24	Penicillium_sp.	Eurotiales (A)	18	0.8	21.2	10.1	**62.3**	6.4

Table S5: Relative importance of different factors (company 1- 4) and *in vitro* effect variables (TLR4, TLR2) on the fungal community composition from recycling plants, as revealed from PERMANOVA.

Variables	Df	Sums Of Squares	Mean Squares	F. Model	R2	Pr(>F)
Company	3	1.3152	0.4384	5.96	0.44	**<0.001**
TLR4	1	0.2946	0.2946	4.00	0.10	**<0.001**
TLR2	1	0.2247	0.2247	3.05	0.08	**0.003**
Company* TLR4	3	0.3881	0.1294	1.76	0.13	**0.019**
Company* TLR2	3	0.3046	0.1016	1.38	0.10	0.117
Residuals	6	0.4416	0.0736	0.15		
Total	17	2.9688	1.0000			

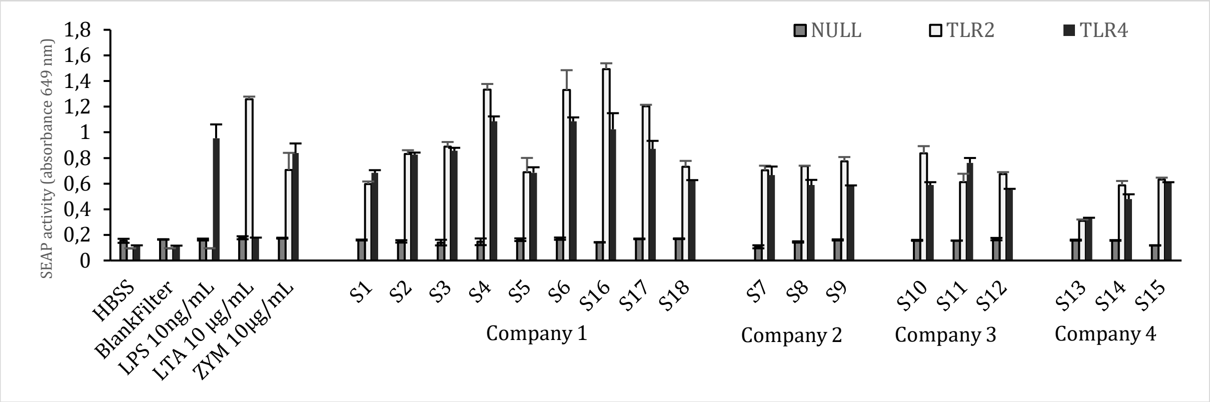

Figure S1: Bar graph of the absorbance measured during the activation of the Null control cells (NULL), TLR2 and TLR4 reporter cells with different controls and dust samples from waste management workers. LPS (lipopolysaccharide), LTA (Lipoteichoic acid) and ZYM (Zymosan) were used as positive controls for TLR4, TLR2 and both, respectively. Data represent the arithmetic mean and the standard deviation of the absorbance from 3 treatments in single experiment.

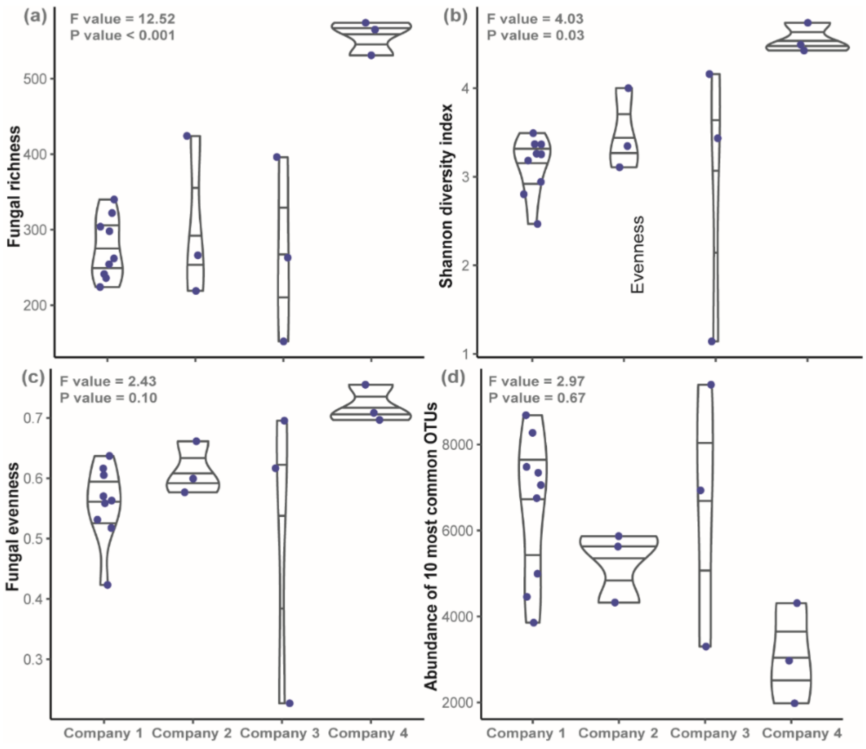

Figure S2: Fungal richness and Shannon diversity index variability among four recycling plants (company 1-4) shown with violin plots. Richness (F=12.52; p value <0.001); Shannon diversity index (F=4.04, p value=0.03). Statistically significant differences among studied factor variables were analyzed using ANOVA and Tukey´s HSD post-hoc test.

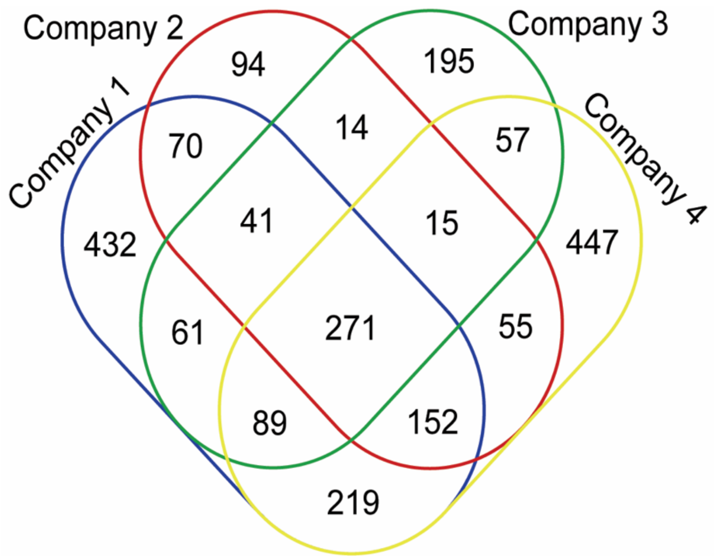

Figure S3: Number of shared and unique OTUs of the fungal composition among four different waste recycling plants.

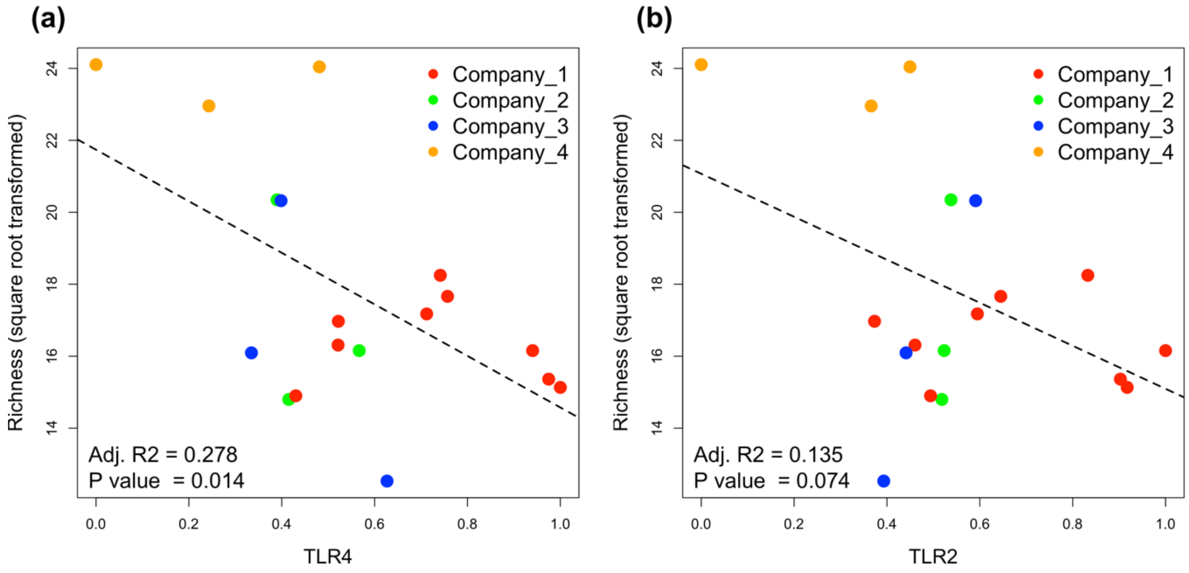

Figure S4: Linear regression analyses between fungal species richness and (a)TLR2 and (b) TLR4 activation.

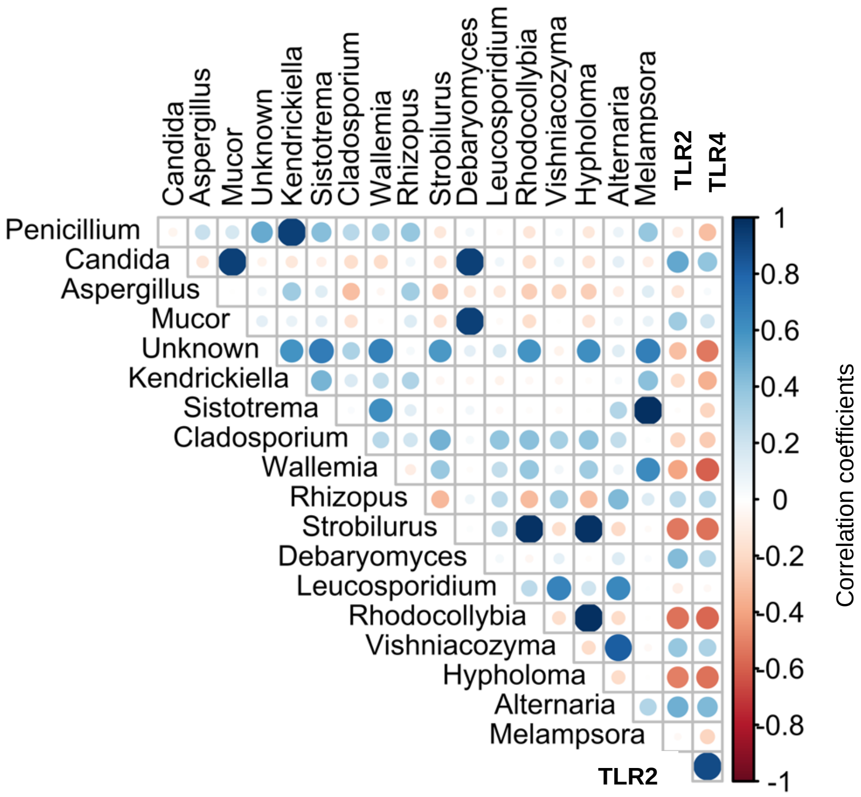

Figure S5 Pearson´s correlation analyses between relative abundance of most common top 20 genera and TLRs
